# Movie Scene Event Extraction with Graph Attention Network Based on Argument Correlation Information

**DOI:** 10.3390/s23042285

**Published:** 2023-02-17

**Authors:** Qian Yi, Guixuan Zhang, Jie Liu, Shuwu Zhang

**Affiliations:** 1 Beijing Engineering Research Center of Digital Content Technology, Institute of Automation, Chinese Academy of Sciences, Beijing 100038, China; 2 School of Computer and Control Engineering, University of Chinese Academy of Sciences, Beijing 100038, China

**Keywords:** event extraction, graph attention network, argument correlation information

## Abstract

Movie scene event extraction is a practical task in media analysis, which aims at extracting structured events from unstructured movie scripts. However, although there have been many studies regarding open domain event extraction, there have only been a few studies focusing on movie scene event extraction. Specifically aimed at instances where different argument roles have the same characteristics in a movie scene, we propose the utilization of the correlation between different argument roles, which is beneficial for both movie scene trigger extraction (trigger identification and classification) and movie scene argument extraction (argument identification and classification) in event extraction. To model the correlation between different argument roles, we propose the superior role concept (SRC), a high-level role concept based upon the ordinary argument role. In this paper, we introduce a new movie scene event extraction model with two main features: (1) an attentive high-level argument role module to capture SRC information and (2) an SRC-based graph attention network (GAT) to fuse the argument role correlation information into semantic embeddings. To evaluate the performance of our model, we constructed a movie scene event extraction dataset named MovieSceneEvent and also conducted experiments on a widely used dataset to compare the results with other models. The experimental results show that our model outperforms competitive models, and the correlation information of argument roles helps to improve the performance of movie scene event extraction.

## 1. Introduction

Event extraction (EE) is an essential task in information extraction. Movie scene event extraction is a practical task in media analysis, which can help viewers to understand the movie plot. In movie scene event extraction, after the event trigger and its argument are obtained from unstructured text, a predefined event type and a role are then assigned to the trigger and the argument, respectively. For example, in the sentence “Peter picks up the pistol and shoots Ruth.”, the predefined event type “Exchange of Fire” and its corresponding trigger “shoots” are first extracted by the trigger extraction module. The argument extraction module needs to further extract the argument subjects, “Peter” and “Rose”, and their argument roles, “Attacker” and “Victim”.

Since event extraction plays a vital role in many downstream tasks, great efforts have been made to improve the performance of event extraction. Conventional event extraction models utilized handcraft features and adopted kernel-based methods [1,2,3,4,5]. However, with more and more attention being paid to deep learning, the application of distributional representation-based methods became more and more popular and achieved better performance [6,7,8,9]. Some recent works have proposed advanced methods to further improve event extraction, for instance, the question answering (QA) framework, with more external information being adopted [10,11].

However, open domain event extraction has attracted a lot of attention, and there is not much research that specifically focuses on movie scene event extraction. In movie scene event extraction, it is not uncommon for different argument roles to have some similar attributes. Take the sentence “Peter picks up the pistol and shoots Ruth.”, for example. The argument roles of “Peter” and “Ruth” are “Attacker” and “Victim”, respectively, and both of these argument roles stem from the SRC “person”. This correlating information between argument roles not only benefits the argument extraction, but also the trigger extraction. Intuitively, the SRC also makes it easier to classify the argument role. For instance, if we know the “Attacker Job” belongs to the SRC “person”, it is easier to classify it into the role of “Attacker”. SRC information also helps to identify the trigger and its event type. For example, for the trigger type “Exchange of Fire”, its arguments usually involve people. So, if we know that the respective candidate arguments are both an SRC “person”, as given in the sentence, the trigger type of the model will tend to be classified as “Exchange of Fire”.

In order to obtain the correlation information between argument roles and further improve movie scene event extraction, we proposed a novel, argument correlation, information-based GAT. To capture the correlation information between argument roles, an attentive high-level argument role module is applied to obtain SRC features. Then, the GAT [12] is employed to extract semantic features via a dependency tree, which is able to link related mentions and shorten the distance between the trigger and its arguments. In addition, the attention unit in the GAT is utilized to integrate SRC information into the semantic features.

In addition, because there is no special dataset for movie scene event extraction, the lack of professional datasets has also become an urgent problem that requires a solution. In order to further explore movie scene event extraction and solve this problem, we constructed an event extraction dataset for movie scenes. We choose movie scene sentences from the film scripts and labeled them manually. The dataset contains 5852 training samples and 486 testing samples, with 12 event types and 18 argument roles. This dataset helps us further verify the effectiveness of our algorithm in the task of movie scene event extraction.

In this paper, our main contributions are as follows:

- We introduce the correlation information of argument roles to further improve joint movie scene event extraction.
- We propose an SRC-based GAT to capture the semantic features and integrate the correlation information of argument roles into the semantic features.
- We constructed a movie scene extraction dataset to verify the effectiveness of our model. The experimental results show that our model outperforms competitive models, and the correlation information between argument roles can help to improve the performance of movie scene event extraction.

The remainder of this paper is organized as follows. In Section 2, we present related work concerning event extraction. In Section 3, we outline our proposed relation extraction model. Next, in Section 4, we present the experimental results of our model and then analyze the results. Finally, in Section 5, we give the conclusions of our paper and introduce our future work.

## 2. Related Work 

Natural language processing (NLP) technology is widely used in media analysis [13,14,15,16]. As an essential task of NLP, event extraction can be divided into two subtasks: (1) event trigger extraction, in which the “trigger” (that represents the occurrence of an event) is extracted and then assigned an event type, and (2) argument extraction, in which the arguments of the trigger are detected, and then each argument is assigned an argument role with respect to the event type.

Earlier works paid more attention to the pipeline methods or one of the subtasks. Two types of efforts can be identified. One relies on hand-crafted features [1,2,3,4,5,17]. For instance, Mcclosky [1] et al. utilized the tree of event–argument relations as features for event extraction. Liao et al. [2] and Ji et al. [4] used the document-level features and Li et al. [3] used global features to improve performance, while Huang et al. [5] modeled the textual cohesion of the text as features. The other is the neural network method [7,8]. Nguyen et al. [7], for example, utilized graph convolutional networks (GCNN) for event detection, and in [8], they adopted domain adaptation convolutional neural networks (CNN) to improve event detection. 

However, as error propagation problems are present in these works, the accuracy of downstream subtasks will be impacted. Thus, more and more joint models have been proposed to solve this problem [18,19,20]. Chen et al. proposed the dynamic multi-pooling CNN model to extract semantic features [9]. Sha et al. [6] utilized recurrent neural networks (RNN) to embed a dependency bridge, which achieved good performance. Li et al. [21] introduced external knowledge to improve domain-specific event extraction. Recently, some works have adopted a generative method [22,23] to solve event extraction problems. QA frameworks have been adapted for event extraction and can also effectively solve the problem of few-shot event extraction [10,11].

However, the above methods are mainly aimed at open domain event extraction tasks and do not take the characteristics of movie scene event extraction into consideration. In this paper, considering that argument roles in movie scene event extraction usually belong to several specific categories, we define several SRCs to model the correlation between different argument roles when proceeding with joint event extraction. Furthermore, we designed two specific modules to utilize the correlation information to improve the performance of the two subtasks of event extraction.

## 3. Model

**Figure 1:**
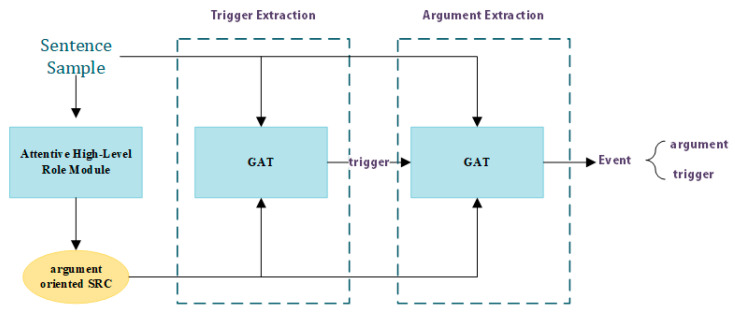
The process of movie scene event extraction.

In this section, we introduce our model for movie scene event extraction in detail. The whole process of movie scene event extraction is shown schematically in Figure 1. As Figure 1 shows, the whole process can be divided into three steps: (1) obtaining the argument-oriented SRC embedding through the attentive high-level role module; (2) incorporating the argument-oriented SRC embedding into GAT for trigger extraction; and (3) incorporating the argument-oriented SRC embedding into GAT for argument extraction. In the remainder of this section, we first present how to obtain argument-oriented SRC embedding through an attentive high-level role module, then we introduce the framework of GAT. Finally, we demonstrate how to incorporate the argument-oriented SRC embedding into GAT and apply it for trigger extraction and argument extraction.

### 3.1. Attentive High-Level Role Module

**Figure 2:**
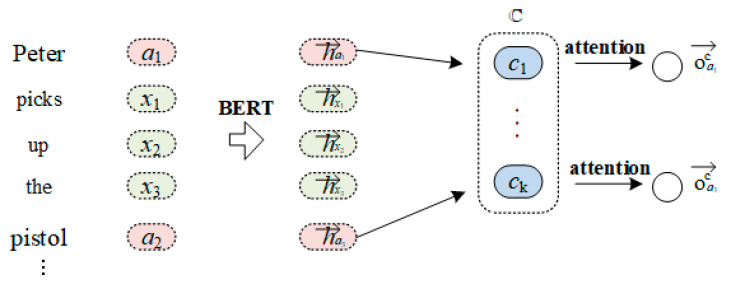
Framework of attentive high-level role modules.

Figure 2 shows the structure of the attentive high-level role module, and we introduce it in detail here.

The input sentence can be denoted as a n-word sequence *s = {x_*1, *x_*2, …, *a_*1, …, *a_k*, …, *x_(n − k)}*, in which *a_i* denotes the candidate argument. The candidate arguments are the named entities extracted by StanfordNER [24]. To obtain the hidden embedding of each word, we employed BERT [25] as the encoder, which achieved the STOA performance on a wide range of NLP tasks. So, we can embed the members of the word sequence into their hidden representations:

(1) $$\left\{h_{1},h_{2},\ldots ,h_{a1},\ldots ,h_{ak},h_{n-k}\right\}=BERT\left(x_{1},x_{2},\ldots ,a_{1},\ldots ,a_{k},\ldots ,x_{n-k}\right)$$

where $h_{i}\in R^{d}$ and d are the word embedding sizes. 

In terms of candidate argument $a_{i}$, different SRCs have different degrees of correlation with it. Firstly, for each SRC c, we assign a trainable embedding vector $u_{c}\in R^{d}$. Then, with respect to the given candidate argument $a_{i}$, we calculate the attention score $s_{a_{i}}^{c}$ of each SRC *c*:

(2) $$h_{a_{i}}^{c}=ReLU\left(W_{a}\cdot \left[u_{c}||h_{ai}\right]\right);$$

(3) $$s_{a_{i}}^{c_{j}}=\frac{exp\left(W_{b}\cdot h_{a_{i}}^{c_{j}}\right)}{\sum_{C}exp\left(W_{b}\cdot h_{a_{i}}^{c_{k}}\right)},$$

where $W_{a}\in R^{d\times 2d}$ and $W_{b}\in R^{d}$ are the weighted matrices, || denotes the concatenation operation through the dimensions, and C is the set of candidate superior role concepts. Finally, we are able to obtain the argument-oriented SRC embedding vector $o^{C}\;$ by calculating the weighted sum of the embedding vectors of superior role concepts:

(4) $$o_{a_{i}}^{C}=\sum_{c\in C}s_{a_{i}}^{c}\cdot u_{c}.$$

Intuitively, $o_{a_{i}}^{C}$ contains the semantic information of the SRC that has a stronger correlation with the candidate argument *a_i_*.

### 3.2. Event Trigger Extraction

**Figure 3:**
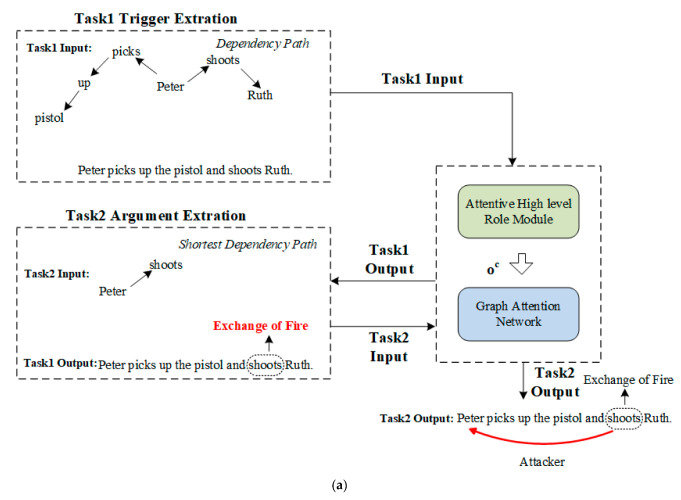

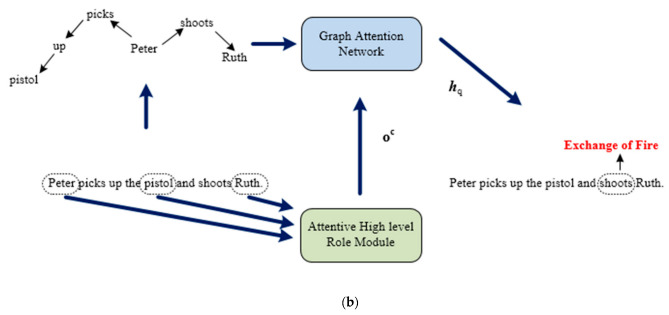
(**a**) The overall framework of the whole event extraction model; (**b**) the details of trigger extraction.

Figure 3 shows an example of the whole event extraction process and the details of trigger extraction. In this section, we introduce the details of the latter process.

**The Graph Attention Network** The GAT employs the attention mechanism to embed the tree-structured topology, as its name suggests. It shows improvements in capturing semantic features when compared to conventional sequential models. GAT can be seen as an extension of memory networks [26].

**Figure 4:**
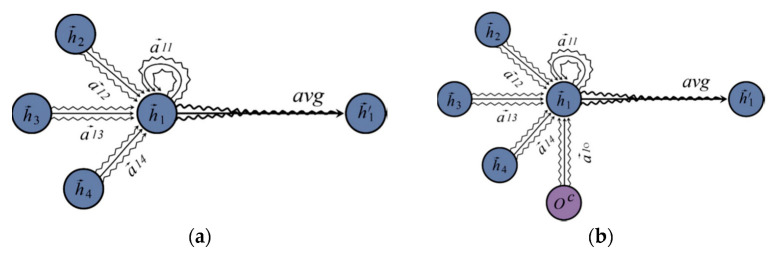
(**a**) An original graph attention unit; (**b**) an SRC-based graph attention unit. The purple circle labelled $o^{c}$ represents the SRC features.

For each node in GAT, their hidden states are obtained by using the attention unit to calculate the weighted sum of the hidden states of their children nodes in the graph structure. Figure 4a shows the operation of one node in GAT. For each node on the dependency tree, $S\left(q\right)$ denotes the set of children nodes of node q. We can obtain the corresponding attention score using the following equation:

(5) $$\alpha_{qj}=\frac{exp\left(LeakyReLU\left(h_{j}\cdot W_{g}\cdot h_{q}\right)\right)}{\sum_{k\in S\left(q\right)}exp\left(LeakyReLU\left(h_{k}\cdot W_{g}\cdot h_{q}\right)\right)},$$

where node j  is the child node of q, and $W_{q}\in R^{d\times d}$ is the weighted matrix.

Then, we obtain the embedding $h_{q}$ of q by calculating the weighted sum of the hidden embedding of $S\left(q\right)$:

(6) $$h_{q}=\sigma \left(\sum_{k\in S\left(q\right)}\alpha_{qj}\cdot h_{k}\right).$$

where σ represents the sigmoid function, and $\;h_{k}$ is the hidden embedding of node k. 

In order to obtain the hidden embedding of node j, the graph attention unit calculates all of its children nodes’ hidden states through depth-first traversal [27].

**Superior Role Concept-Based Graph Attention Network** Utilizing Formulas (1)–(4), we are able to obtain the argument-oriented SRC embedding $o_{a}^{C}$. Specifically, for each candidate argument, we obtain the correlation between each argument word by calculating the attention score of each SRC embedding $u_{c}$. Finally, we can obtain an argument-oriented SRC embedding vector $o_{a}^{C}$ by calculating the weighted sum of $u_{c}$. It is worth noting that we use the full set of all superior role concepts C to calculate the argument-oriented SRC embedding vector during trigger extraction, since, in trigger extraction, we do not know the trigger and the event type and, therefore, have not obtained their corresponding argument roles.

After the argument-oriented SRC embedding $o_{a}^{C}$ is obtained, we then further incorporate it into the GAT to use the SRC information to improve the trigger extraction. Figure 4b demonstrates how to fuse the argument-oriented SRC embedding into GAT.

For a given sentence, we first utilize the Stanford Parser [24] to obtain its dependency tree, and the tree structure will then be fed to the GAT. For each node q on the dependency tree, $S\left(q\right)$ denotes the set of children nodes of node q, and $o_{q}^{C}$ is the SRC information embedding of q. $o_{q}^{C}$ is set to 0 if q is not a candidate argument. Normally, in the original GAT, we calculate the attention score of each node j in *S(q)* directly. However, to merge the extra-high-level argument role information, we treat the embedding vector $o_{q}^{C}$ as a child node of q, so we are able to obtain the corresponding attention score:

(7) $$\alpha_{qj}=\frac{exp\left(LeakyReLU\left(h_{j}\cdot W_{g}\cdot h_{q}\right)\right)}{\sum_{k\in S\left(q\right)\cup \left\{o\right\}}exp\left(LeakyReLU\left(h_{k}\cdot W_{g}\cdot h_{q}\right)\right)},$$

where node  j  can be either the child node of q or the argument-oriented SRC embedding vector of q. When q is not a candidate argument node, the attention score of o is 0.

Then, we obtain the embedding of q by calculating the weighted sum of the hidden embedding of $S\left(q\right)$ and the argument-oriented SRC embedding vector $o^{c}$:

(8) $$h_{q}^{\prime }=\sigma \left(\sum_{k\in S\left(q\right)\cup \left\{o\right\}}\alpha_{qj}\cdot h_{k}\right).$$

where σ represents the sigmoid function. To capture more affluent features, a multi-head attention is applied. So, the final embedding of q can be obtained from the expression below:

(9) $$h_{q}^{\prime }=\sigma \left(\frac{1}{M}\overset{M}{\underset{m=1}{\sum }}\underset{k\in S\left(q\right)\cup \left\{o\right\}}{\sum }\alpha_{qj}\cdot h_{k}\right)$$

where *M* is the number of attention heads; here, we adopt a three-head attention. After the hidden state of each node is obtained, we send the embedding to a feed forward layer with a SoftMax classifier in order to predict the trigger type for each node and optimize the parameters by minimizing a negative log-likelihood loss. 

### 3.3. Event Argument Extraction

**Figure 5:**
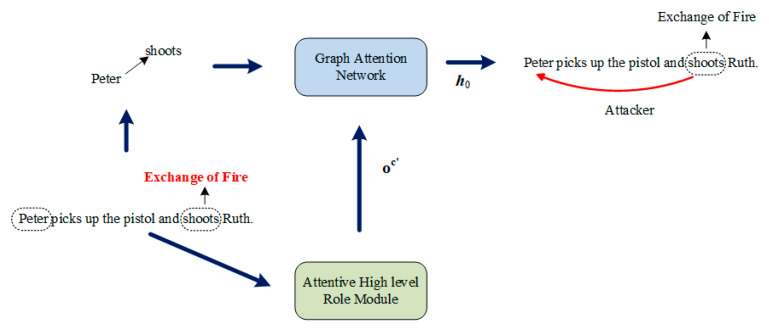
The details of the event argument extraction.

After all candidate event triggers are obtained after trigger extraction, we begin extracting event arguments with respect to the given triggers. Figure 5 shows the details of the event argument extraction module.

Unlike trigger extraction, we adopt the shortest dependency path (SDP) as the input for SRC information-based GAT in argument extraction, as SDP can better capture the correlation between the trigger and argument. Figure 4 shows an example of SDP and its corresponding dependency tree—it is easy to see that SDP is more concise, as redundant information can be eliminated.

Another difference between argument extraction and trigger extraction is that, in argument extraction, we know the trigger and the event type. So, when calculating the argument-oriented SRC embedding $o^{c}$ in the argument extraction procedure, we only utilize the corresponding SRC of the given event type to calculate $o^{c}$. For instance, for the event type “Exchange of Fire”, its argument roles stem from three role concepts: person, place, and item. Thus, when calculating the argument-oriented SRC embedding for the arguments of the event type “Exchange of Fire”, following Formulas (2)–(4), the set of candidate superior role concepts is *C* = {Person, Place, Item}.

Specifically, given an event type and its candidate argument, its corresponding SRC set is $C^{\prime }=\left\{c_{1},c_{2},\ldots ,c_{k}\right\}$, where k is the number of SRCs corresponding to the given event type. Following Formulas (1)–(4), we are able to obtain the argument-oriented SRC embedding $o_{a}^{C\prime }$. We then utilize the SRC-based GAT introduced in the event trigger extraction module to encode each node in the SDP into a new hidden state representation and use the hidden embedding of the root node $h_{0}$ as the embedding vector of the input. For each candidate argument, we can obtain an argument-role-oriented instance embedding $h_{0}$.

Then, we use the argument-role-oriented instance embedding $h_{0}$ as the input feature for argument role classification and send the embedding to a feed forward layer with a SoftMax classifier to predict the argument role. We optimize the parameters by minimizing a negative log-likelihood loss. 

## 4. Experiments

### 4.1. Experiment Setup

**Datasets**

1. MovieSceneEvent: We constructed a movie scene event extraction dataset named MovieSceneEvent for this research. To construct a movie-scene-specific event extraction dataset, we first summarized 12 common types of events based on the research needs and the suggestions of professionals in the film field. Then, we chose sentences related to these events from movie script texts. These movie scripts were selected from 13 common genres of movies (including romance, comedy, action, war movies, and so on). According to the defined event types, we first used the manually defined template to roughly screen out the texts related to the defined event type from the script text and then manually filter these texts. Finally, these sentences were further labeled manually. We asked two annotators to label each sample. If their labeling was consistent, that result was used for the sample. If not, a third annotator was used to ensure the accuracy of the labeling. The movie scene event extraction dataset contains 5852 training samples and 486 testing samples, with 12 event types and 18 argument roles.
2. ACE2005: Following previous works [3,28], we also adopted ACE2005, the widely used event extraction dataset, to evaluate the effectiveness of our model. It contains 599 documents, with 13,672 labeled sentences in the ACE2005 dataset, and these sentences are labeled with 8 given event types, 33 event subtypes, and 35 argument roles. Following [3,26], we split the ACE2005 dataset into 529, 30, and 40 documents for training, development, and testing, respectively.

**Evaluation Measures** Following previous work [6,7,8,9], we evaluated our model on four metrics: (1) trigger detection: a trigger is considered correctly detected if span offsets correctly match the label; (2) trigger classification: a trigger is considered correctly classified only if both of its span offsets and event subtypes are correct; (3) argument detection: an argument is considered correctly detected if its span offsets match the label and its event subtypes exactly match the label; and (4) argument classification: an argument is considered correctly classified only if its span offsets, event subtypes, and argument roles are correct. For example, in the sentence “Peter picks up the pistol and shoots Ruth.”, for the first metric, the trigger word “shoots” is considered correctly detected if span offsets correctly match the label “(6,1)”, in which “6” indicates the start index and “1” indicates the span. For Metric 2, the trigger “shoots” is considered correctly classified only if both of its span offsets “(6,1)” and the event subtype are correctly classified as “Exchange of Fire”. For Metric 3, the argument “Peter” is considered correctly detected if its span offsets match the label “(0,1)” and its event subtype exactly matches the label “Exchange of Fire”. For Metric 4, the argument “Peter” is considered to be correctly classified only if its span offsets (“(0,1)”), event subtype (“Exchange of Fire”), and argument role (“Attacker”) are correct. All experimental results are presented in the form of precision (P), recall (R), and F-measure ($F1=2*P*R/\left(P+R\right)$) for each metric. 

**The definition** of the superior role concept is based on experience; thus, we define four superior role concepts manually: person, place, item, and time. The results of superior role concepts in this paper cannot be directly extended to other datasets with different label definitions, but the definition method is simple. 

**Figure 6:**
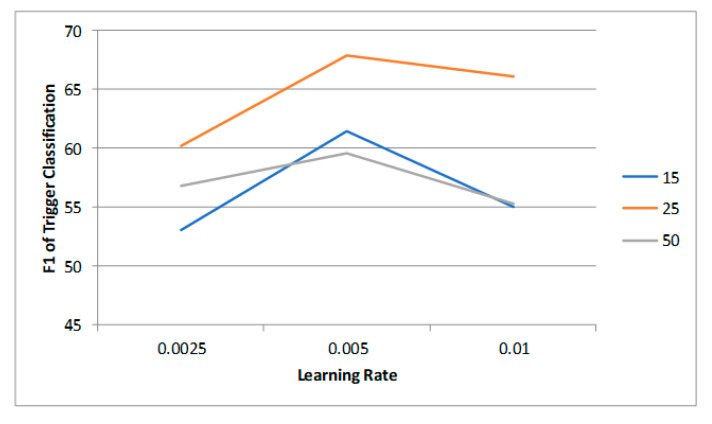
The influence of learning rate and batch size on trigger classification.

**Figure 7:**
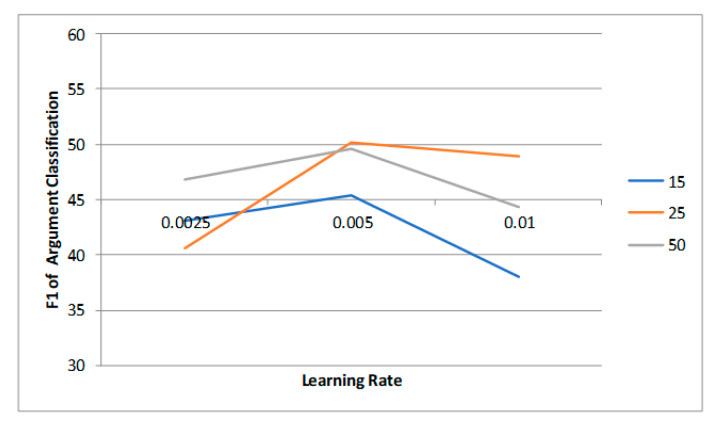
The influence of learning rate and batch size on argument classification.

**Hyperparameters** We used BERT for sequence encoding to obtain the hidden embeddings and adopt the BERT–BASE–CASED [25] model. As for the hyperparameters, we tried the following parameter settings: learning rate = {0.0025, 0.005, 0.01}; batch size = {15, 25, 50}. The experiment results of different settings of parameters on the MovieSceneEvent dataset are shown in Figure 6 and Figure 7. We ultimately chose 0.005 and 25 as the learning rate and batch size, respectively. The hyperparameters are listed in Table 1. We utilized two NVIDIA K40 as the running environment, and the details of the model training are also listed in Table 1.

### 4.2. Overall Performance 

We compared the performance of our model with several representative models. **JOINTFEATURE** [28] considered the relationship between triggers, arguments, and their correlations and conducted joint inference of these variables across a document. **dbRNN** [6] adopted LSTM as encoder to embed the dependency tree structure in order to extract the event trigger and argument role. **Joint3EE** [7] conducted the event extraction in a multi-task module with shared BiGRU hidden representations. **BS** [10] is a representative model-adapted QA framework, which used bleached statements (BSs) to give a model access to information contained in annotation manuals. **Text2Event** [29] is a sequence-to-structure generation model that can directly extract events from the text in an end-to-end manner.

Table 2 shows the comparison between our model and other models using the movie scene event extraction dataset. Table 3 shows the comparison between our model and the above models on an open domain event extraction dataset. Table 2 and Table 3 demonstrate that our model achieves the best performance on most of the evaluation criteria, especially on the F1 scores in the classification results. Specifically, in the MovieSceneEvent dataset, our model achieves F1 scores of 70.3% on trigger identification, which is 8.2%, 7.8%, 1.2%, 2.1%, and 1.1% higher than that of JOINTFEATURE, dbRNN, Joint3EE, BS, and Text2Event, respectively. Our model achieves F1 scores of 67.3% on trigger classification, which is 7.9%, 11.9%, 3.9%, 2.6%, and 3.2% superior to that of JOINTFEATURE, dbRNN, Joint3EE, BS, and Text2Event, respectively. Our model achieves F1 scores of 53.7% on argument identification, which is 8.3%, 8.5%, 3.8%, 11.1%, and 7.5% higher than that of JOINTFEATURE, dbRNN, Joint3EE, BS, and Text2Event, respectively. Our model achieves F1 scores of 53.7% on argument classification, which is 7.2%, 5.6%, 3.7%, 12.2%, and 2.3% better than that of JOINTFEATURE, dbRNN, Joint3EE, BS, and Text2Event, respectively. On the ACE2005 dataset, our model achieves F1 scores of 73.3% on trigger identification, which is 3.2%, 0.8%, and 0.4% higher than that of JOINTFEATURE, Joint3EE, and BS, respectively. Our model achieves F1 scores of 72.6% on trigger classification, which is 3.9%, 0.7%, 2.8%, 2.1%, and 0.8% higher than that of JOINTFEATURE, dbRNN, Joint3EE, BS, and Text2Event, respectively. Our model achieves F1 scores of 55.7% on argument identification, which is 5.1% and 12.7% higher than that of JOINTFEATURE and BS, respectively, and 1.5% and 4.2% lower than that of dbRNN and Joint3EE, respectively. Our model achieves F1 scores of 54.7% on argument classification, which is 6.3%, 4.6%, 2.6%, 12.3%, and 0.3% better than that of JOINTFEATURE, dbRNN, Joint3EE, BS, and Text2Event, respectively. From the experimental results, we can see the following:

(1) Our model steadily outperforms all other competitive models in both the trigger extraction and argument extraction of movie scene event extraction and open domain event extraction, which indicates that the SRC information can benefit both trigger and argument extraction in event extraction.
(2) In argument extraction, our model significantly outperforms prior work, which may be due to the fact that the SRC information has a more direct correlation with the argument role.
(3) It worth noting that the drop of F1 between both argument identification and classification, as well as trigger identification and classification, is smaller than in previous works, which means the SRC information is able to benefit the classification of both argument role and trigger event types. SRC information helps to maintain more semantic information between identification and classification.
(4) When concerning the performance on open domain datasets, the improvement of our model is much smaller. This is probably due to the composition of argument roles in movie scene event extraction being much easier to generalize into several superior role concepts. Thus, the influence of SRC information is more significant.

### 4.3. Effect of Superior Role Concept

To better understand how the SRC influences our model’s performance, in this section, we conducted an ablation study by adding the SRC into different procedures of the whole model, and Table 4 presents the results of the ablation experiments.

In Table 4, the GAT model shows the removal of the SRC information from both the trigger extraction step and the argument extraction step. It uses GAT to embed the tree structure directly, without any other information. The GAT-TRI+SRC and the GAT-ARG+SRC mean that only the SRC information is introduced into the trigger extraction module and the argument extraction module.

As Table 4 shows, when we adopt the GAT model without any extra information, the performances of the four subtasks drop significantly when compared to the whole model. The F1 scores of the 4 subtasks drop by 3.3%, 1.6%, 2.2%, and 6.6%, respectively. This indicates that the correlation information between argument roles is beneficial to both the trigger extraction and the argument extraction.

However, the situations are different when the SRC information is removed from trigger extraction and argument extraction. The improvement was not obvious when only SRC information was added to the trigger extraction module, although the argument extraction performance was improved when only SRC information was added to the argument extraction module. We think this situation arises not because the high-level role information is not useful for trigger extraction, but because the hierarchical relationship between the SRC and the argument role can only be updated through the argument extraction module. In other words, the SRC embedding vectors are mainly trained in the argument extraction module.

### 4.4. Influence of Dataset Size

We also explored how the size of the dataset influences the performance of our model. We selected 75%, 50%, and 25% samples from the training data to train our model. These samples were selected randomly and uniformly. The results are given in Table 5.

From the experimental results, we can see that when the size of the dataset is reduced to 75%, the performance of the model only decreases slightly. However, when the size of the dataset is reduced to 50%, or even 25%, the performance of the model decreases significantly. When the size of the dataset is reduced to 25%, the F1 scores of the trigger and argument classification drop by 35.1% and 26.0%, respectively. When the size of the dataset is reduced to 75%, the F1 scores of the trigger and argument classification only drop by 2.4% and 2.2%, respectively. Therefore, the size of the dataset has a significant impact on the effect of the model. However, when the dataset reaches a certain size, the impact of increasing the size of the training set on the results gradually decreases.

## 5. Discussion

In this paper, we proposed an argument correlation, information-based, graph attention network for movie scene event extraction. Specifically, in order to verify the effectiveness of the proposed model, we compared and discussed the performance of the five models JOINTFEATURE, dbRNN, Joint3EE, BS, and Text2Event on two datasets, MovieSceneEvent and ACE2005. The experimental results demonstrate that our models steadily outperform all other competitive models, regarding both trigger extraction and argument extraction, on movie scene event extraction and open domain event extraction. However, when compared with the performance in the open domain dataset, the improvement of our model in the movie scene event extraction dataset is much more significant. This is probably due to the composition of argument roles in movie scene event extraction being much easier to generalize into several superior role concepts. Moreover, the ablation study in Section 4.3 verifies that that the correlation information between argument roles is beneficial to both the trigger extraction and the argument extraction. Furthermore, the study regarding the influence of dataset size indicates that this factor has a significant influence on the performance when the size of the dataset decreases by a significant amount. However, when the dataset reaches a certain size, the impact of increasing the size of the training set on the results is not significant.

## 6. Conclusions

In this paper, we propose an argument correlation, information-based movie scene event extraction model because existing open domain event extraction methods have not paid attention to the specific information in a certain field nor made full use of the correlation information of argument roles, which is important implicit semantic information in movie scene event extraction. In order to fully utilize this important implicit semantic information to improve movie scene event extraction, we design an SRC-based GAT to capture this implicit information and integrate correlation information of argument roles into the semantic features. The GAT module can capture the semantic features through the dependency tree structure, while fusing the SRC information into the nodes’ hidden embedding. In order to verify the effectiveness of our model, we constructed a movie scene event extraction dataset. Experimental results show that the SRC helps to improve the performance of both event trigger extraction and argument extraction. Our model can significantly improve the performance of movie scene event extraction. Meanwhile, it also has good performance in open domain event extraction.

In the future, we will further exploit the influence of external information on event extraction. We will also attempt to integrate our model with recently popular structures, such as a QA framework.

## Figures and Tables

**Table 1:** Hyperparameters.

Parameter	Value
Word embedding size d	768
Batch size	25
Epoch size	20
Dropout rate	0.5
Learning rate	0.005
Optimizer	AdaGrad

**Table 2:** Results from the MovieSceneEvent dataset.

Model	Trigger						Argument					
	Identification			Classification			Identification			Classification		
	P	R	F1	P	R	F1	P	R	F1	P	R	F1
JOINTFEATURE	61.0	63.2	62.1	70.1	51.6	59.4	51.0	40.9	45.4	44.3	41.6	42.9
DbRNN	63.3	61.8	62.5	61.1	50.7	55.4	41.7	49.5	45.2	43.5	45.6	44.5
Joint3EE	65.8	72.9	69.1	60.5	66.7	63.4	48.9	51.1	49.9	50.7	42.8	46.4
BS	66.4	70.8	68.2	61.7	68.1	64.7	42.0	43.3	42.6	40.1	35.9	37.9
Text2Event	68.2	70.3	69.2	62.1	66.2	64.1	45.3	47.2	46.2	47.3	48.4	47.8
Ours	69.1	71.6	70.3	65.6	69.1	67.3	50.6	57.3	53.7	53.3	47.3	50.1

**Table 3:** Results from the ACE2005 dataset.

Model	Trigger						Argument					
	Identification			Classification			Identification			Classification		
	P	R	F1	P	R	F1	P	R	F1	P	R	F1
JOINTFEATURE	77.6	65.4	70.1	75.1	63.3	68.7	73.7	38.5	50.6	70.6	36.9	48.4
dbRNN	-	-	-	70.1	69.8	71.9	-	-	57.2	-	-	50.1
Joint3EE	70.5	74.5	72.5	68.0	71.8	69.8	59.9	59.8	59.9	52.1	52.1	52.1
BS	68.9	77.3	72.9	66.7	74.7	70.5	44.9	41.2	43.0	44.3	40.7	42.4
Text2Event	-	-	-	71.2	72.5	71.8	-	-	-	54.0	54.8	54.4
Ours	70.4	76.6	73.3	70.2	75.1	72.6	58.4	53.3	55.7	56.7	52.8	54.7

**Table 4:** Effect of SRC.

Model	Trigger						Argument					
	Identification			Classification			Identification			Classification		
	P	R	F1	P	R	F1	P	R	F1	P	R	F1
GAT	65.8	68.3	67.0	66.7	64.8	65.7	50.9	52.1	51.5	45.1	42.1	43.5
GAT-TRI+SRC	66.1	65.2	65.6	66.0	62.2	64.0	55.6	51.1	53.2	48.5	40.6	44.2
GAT-ARG+SRC	65.3	72.4	68.6	65.5	70.0	67.7	52.8	50.9	51.8	50.9	47.1	48.9
Ours	69.1	71.6	70.3	65.6	69.1	67.3	50.6	57.3	53.7	53.3	47.3	50.1

**Table 5:** Influence of dataset size.

Size	Trigger						Argument					
	Identification			Classification			Identification			Classification		
	P	R	F1	P	R	F1	P	R	F1	P	R	F1
25%	45.8	32.3	37.9	29.6	35.4	32.2	25.6	21.1	23.1	21.1	28.1	24.1
50%	49.1	47.2	48.1	46.0	52.2	48.9	45.3	49.1	47.1	38.5	42.6	40.4
75%	65.5	68.4	66.9	64.5	70.0	67.1	50.8	52.9	51.8	49.9	46.1	47.9
100%	69.1	71.6	70.3	65.6	69.1	67.3	50.6	57.3	53.7	53.3	47.3	50.1

## Data Availability

The data presented in this study are available from the corresponding authors. The data cannot be made public, as they relate to ongoing projects.
